# Causes of variability in prevalence rates of communicable diseases among secondary school Students in Kisumu County, Kenya

**DOI:** 10.1007/s10389-016-0777-9

**Published:** 2016-12-03

**Authors:** David Otieno Odongo, W. J. Wakhungu, Omuterema Stanley

**Affiliations:** Center for Disaster Management and Humanitarian Assistance, Masinde Muliro University of Science and Technology, Kakamega, Kenya

**Keywords:** Risk factor, Variability, Prevalence, Correlation

## Abstract

**Purpose:**

To determine causes of variability in communicable disease prevalence rates among students in secondary schools to inform policy formulation in the public health sector.

**Methods:**

A representative cluster sample size for students was estimated using Fisher et al.’s formula while schools, sub-counties and education zones were clustered and sample size was calculated based on coefficient of variation by school type. Data were collected by questionnaire, medical examination using standard procedures, and focus group discussion, and descriptive analysis was performed on the completed questions. Comparisons between risk factors were made by chi-square and ANOVA analysis using SPSS for Windows (version 15.2; Chicago, IL) software. A *p* value of < 0.05 was considered statistically significant.

**Results:**

There was significant variation between communicable disease prevalence rates and age (X^2^_4, 0.05_ = 2.458), school size (X^2^_12, 0.05_ = 18.636), gender (X^2^_4, 0.05_ = 5.723) and class of students (X^2^_12, 0.05_ = 15.202), and bed and desk spacing (*p* < 0.05 at 95% CI). However, there was no significant association in prevalence rates between both locality and type of school. There was strong evidence that student age has an effect on prevalence rates. The prevalence rate of malaria was higher in male (14.02%) than female students (6.68%) compared to prevalence of diarrhea, which was higher in female (7.96%) than male students.

**Conclusion:**

This study has revealed that the prevalences of diarrhea, tuberculosis, pneumonia and other respiratory tract infections are lower among female secondary school students than males and that the prevalence of malaria is higher in males than females. Age of secondary school students is a significant vulnerability factor for malaria, diarrhea, tuberculosis and pneumonia, which were the important communicable diseases most prevalent among secondary school students in Kisumu County, Kenya.

## Background

The majority of Kenyans continue to seek treatment in health care facilities for ailments that can be controlled through preventive and promotive measures (WHO 2007), with malaria as the leading cause of morbidity at 30% followed by respiratory diseases (24.5%). Tuberculosis control has been more challenging, with high tuberculosis (TB) prevalence of 319 per 100,000, TB/HIV co-infection prevalence of 53% and a growing threat of multidrug resistance/extra-drug resistance (MDR/XDR) TB (WHO 2008). Overcrowding and intermittent use of antibiotics are some of the challenges facing TB control.

Kisumu County suffers from a high burden of communicable diseases as well as emerging threats. According to the Kenya Demographic and Health Survey by the Government of Kenya (2010), the county has one of the highest HIV/AIDS prevalence rates at 17% higher than the Nyanza^1^ region rate of 15.3% and national rate of 7.4%.

These results have been replicated by a demographic and health survey in Kisumu West^2^ (KWHDSS 2011), which found high levels of HIV/AIDS, diarrhea, malaria, multi-drug resistant TB (MDR-TB) and other communicable diseases with more than half of the population relying on surface water as the main source of drinking water while 42% of the households share toilets and 21% have no toilets.

Being a highly disaster-prone county, floods and drought affect different geographical zones annually with a varying degree of damage to the health infrastructure and people’s health, causing interruption of the access to safe water and collapse of the sanitation infrastructure (Mournie 2011). These facts indicate that many people in Kisumu County, students included, are at risk of contracting communicable diseases such as diarrhea, typhoid, intestinal parasite infections, trachoma and schistosomiasis, among others, which account for millions of school days lost (CDC 2007). A major contributing factor to this burden of communicable disease is inadequate access to safe water and sanitation infrastructures (Boschi-Pinto et al. 2008).

The introduction of free schooling in public schools in Kenya has witnessed increased enrollment in secondary schools with the number rising from 1.1 million in 2008 to 1.85 million in 2012 (GoK 2012a, b), leading to increased student membership in the existing hostels and other social amenities in the schools. At present, there is no national guideline to provide a framework for the transformation of school health service into an integrated county health service, while strategies and interventions to work with other ministries have not been spelled out in Kenya’s Health Sector Strategic Plan, 2012–2017 (GoK 2012a, b). Integration of school health services into National and County health services will ensure timely surveillance, prevention and treatment of communicable diseases in schools.

High school students are a neglected group in which very little research has been done concerning the health challenges they face in schools (Muna 2010). There is a need to determine the causes of variability in communicable disease prevalence rates among students to provide baseline information that can be used by policy makers to develop viable intervention programs to address the communicable disease burden in schools.

## Materials and methods

The study applied mixed methods to collect and analyze public health-related risk factors using a correlational research design.

### Sampling strategy

The first three sub-counties out of the six were purposively selected based on the decreasing value of the coefficient of variation by student gender and type of school. A representative cluster sample size (*n* = 400) for 60,230 students was estimated using Fisher et al.’s formula, while the sample size per sub-county was based on the coefficient of variation by student gender. Thirty percent of schools, 38 out of 129, were selected based on the coefficient of variation by school type. Students in sampled schools were clustered and selected by cluster sampling. Key informants and observation units were purposively sampled, while focus group discussions were sampled by quota.

### Data collection and analysis

A series of data collection tools were used. These included:

1. Use of a questionnaire to collect data on the locality of the school, type of school, size/enrollment of the school, demographic information and morbidity of students. Bed spacing in hostels and desk spacing in classrooms were observed using an observation checklist.
2. Focus group discussion guide used to listen and gather information from different people to corroborate data from the field. Thematic issues included groups’ perceptions of skill-based education and a communicable disease surveillance system. An in-depth interview guide was used for index cases.^3^

Students who did not self-report clinically confirmed illnesses in the questionnaire were taken to the nearest health facility for medical examination. The observed medical examination results of interest were:

- Blood slides testing positive for the malaria parasite;
- A positive culture for *Mycrobacterium tuberculosis* confirming tuberculosis infection. An acceptable sputum specimen had more than 25 leukocytes and fewer than 10 epithelial cells per lower field. The most common pathogens detected were bacteria such as *Streptococcus pneumoniae, Staphylococcus aureus* and *Klebsiella species*. Sensitivity testing was done for positive results;
- Positive rapid urine antigen testing for *Streptococcus* pneumonia;
- Positive stool tests for *Clostridium difficile* for respondents having diarrhea or watery stools. Other tests done were an antigen test for *notavirus*, ova and parasite examinations and antigen tests specific for the parasites *Giardia lambia, Entamoeba, histolyca, Crystosporidium* and *Parvum*.
- A descriptive analysis was performed on completed questions. Comparisons between risk factors were made by chi-square and ANOVA analysis using SAS version 9.3. (Carry, NC, USA). A *p* value < 0.05 was considered statistically significant.

We ensured the study was conducted within the international standard procedures for medical examinations. As a result medical examinations were carried out in health facilities in the neighborhood of the schools and performed by medical professionals. Verbal consent from participants was obtained before any data were collected. Participants were informed about the intention to utilize data collected for the dissertation and then intervention. Research authorization was sought from the School of Graduate Studies at Masinde Muliro University of Science and Technology and the administration of Kisumu County, Kenya.

## Results

There were 400 (31%) respondents, 212 male and 188 female students sampled in 38 schools. Characteristics of respondents, including age, gender and class^4^ showed significant variation, *p* < 0.05; however, there was no significant variation in the religion of respondents. The most predominant student age bracket was 14–17 years, forming 67.8% of the sampled population.

**Fig. 1:**
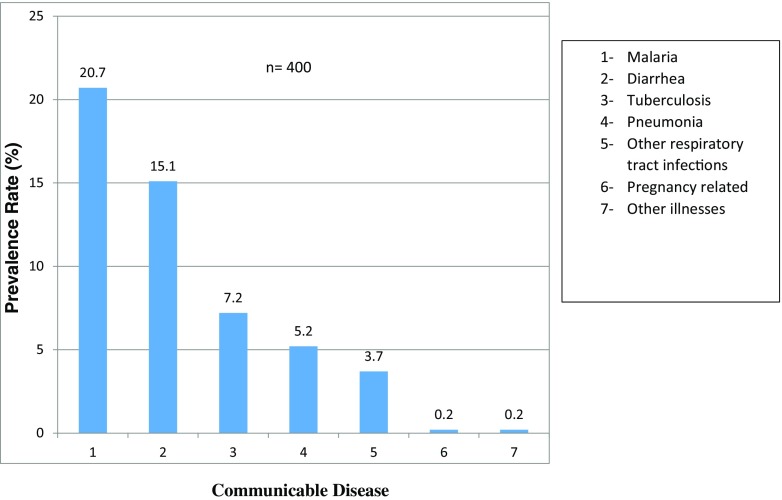
Prevalence of communicable diseases in secondary schools in Kisumu County, Kenya

We observed wide geographical variations in prevalence rates of communicable diseases among schools with the highest being malaria at 20.7% and the lowest being pneumonia at 5.2% (Fig. 1).

**Fig. 2:**
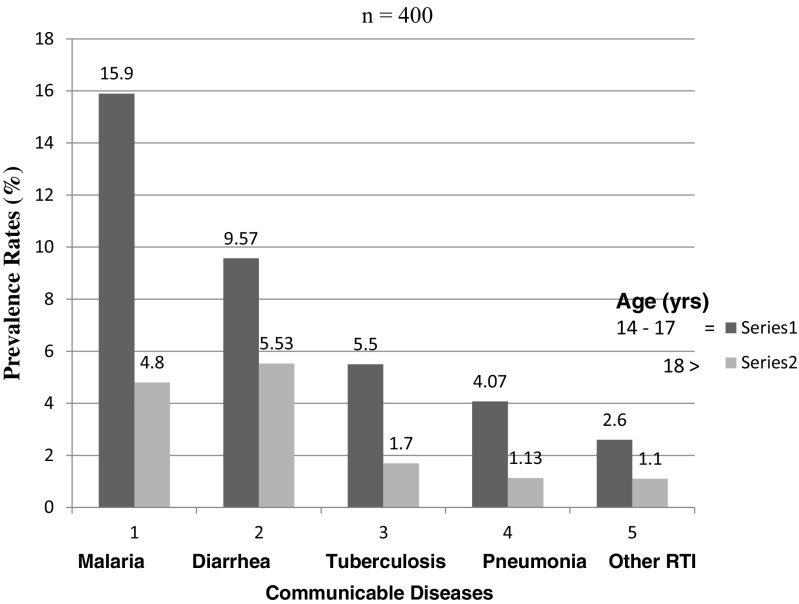
Prevalence rate related to student age

**Fig. 3:**
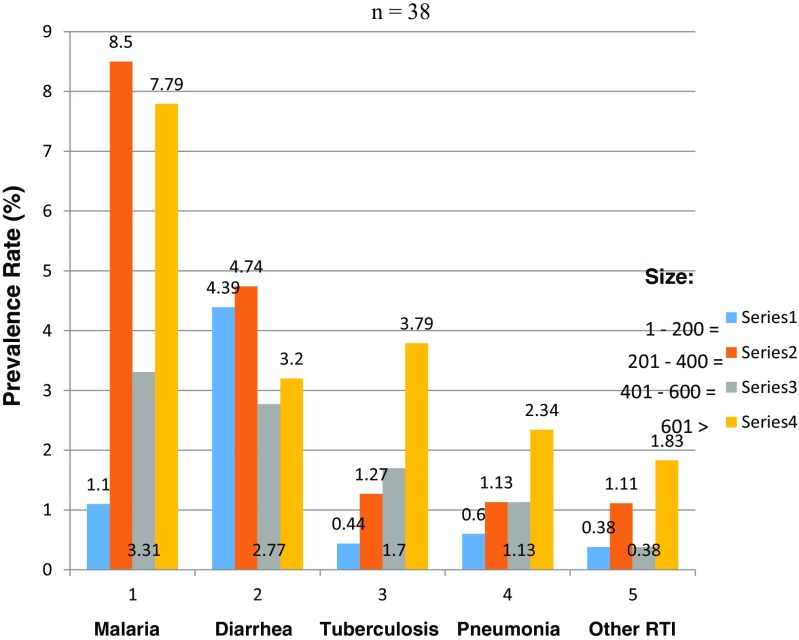
Prevalence rates related to the size/enrollment of schools in Kisumu County

**Fig. 4:**
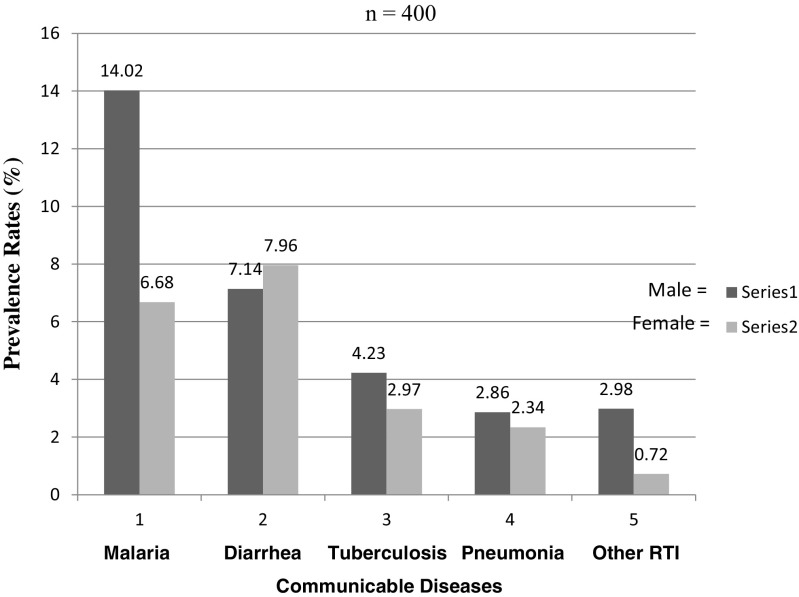
Prevalence rates related to student gender

**Fig. 5:**
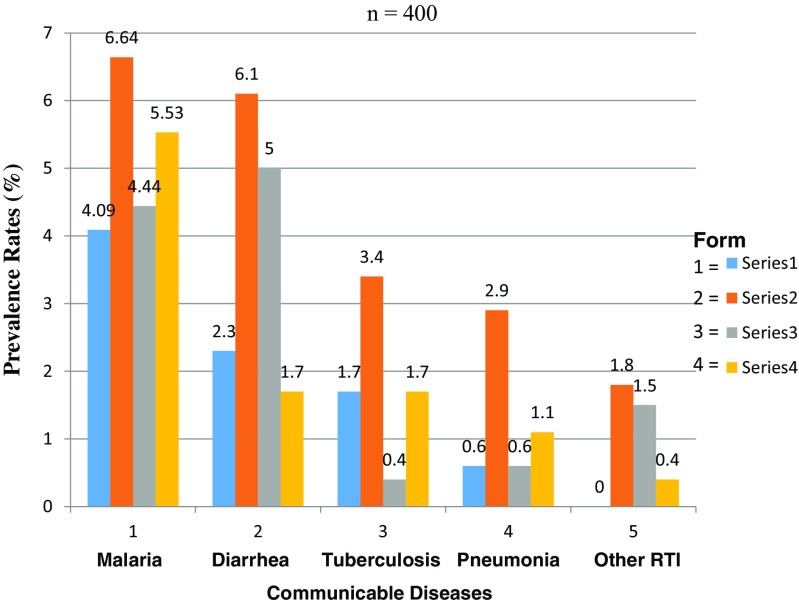
Prevalence rates related to the student class/form

**Fig. 6:**
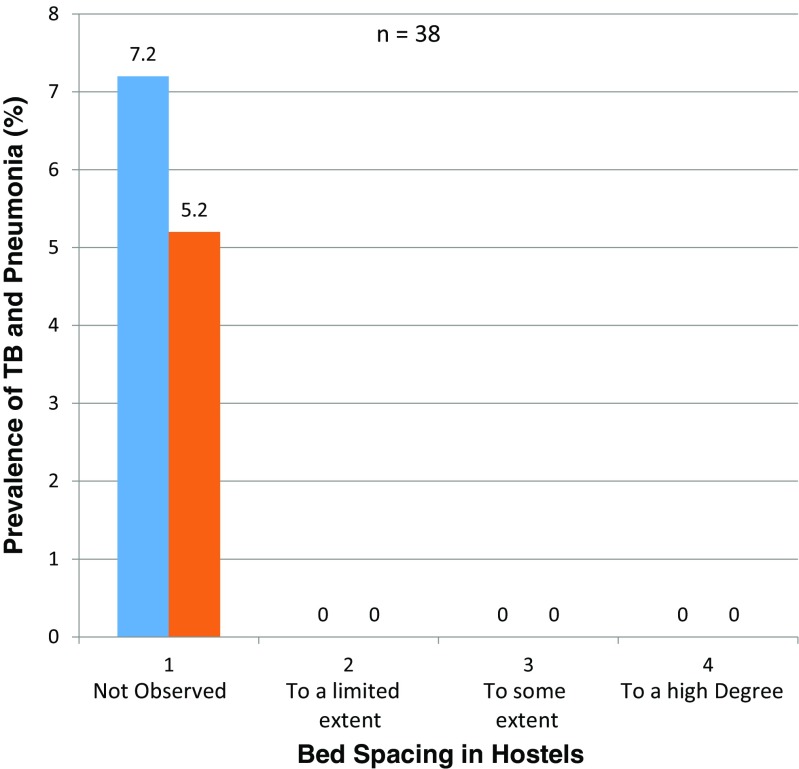
TB and pneumonia prevalence rates related to bed spacing in hostels among secondary schools in Kisumu County, Kenya

There was significant variation^5^ in the prevalence of communicable diseases among secondary schools in Kisumu County. This study found a significant association between the communicable disease prevalence rates and age (X^2^_4, 0.05_ = 2.458, Fig. 2) of students, school size (X^2^_12, 0.05_ = 18.636, Fig. 3)/enrollment, gender (X^2^_4, 0.05_ = 5.723, Fig. 4) and class (X^2^_12, 0.05_ = 15.202, Fig. 5)/form of student, bed spacing in hostels (X^2^_6, 0.05_ = 8.21, Fig. 6) and desk spacing in classrooms.

However, for both the locality and type of school there was no significant association with prevalence rates of communicable diseases. Further analysis using ANOVA showed strong evidence that age has an effect^6^ on prevalence rates of communicable diseases among secondary school students.

The prevalence rates of tuberculosis and pneumonia were 7.2 and 5.2% in schools where standard bed spacing in hostels was not observed and 0% for other observations.

A significant association^12^ between bed spacing and prevalence rates of tuberculosis and pneumonia was observed among students; however, there was no strong evidence that the intervention had an effect.

**Fig. 7:**
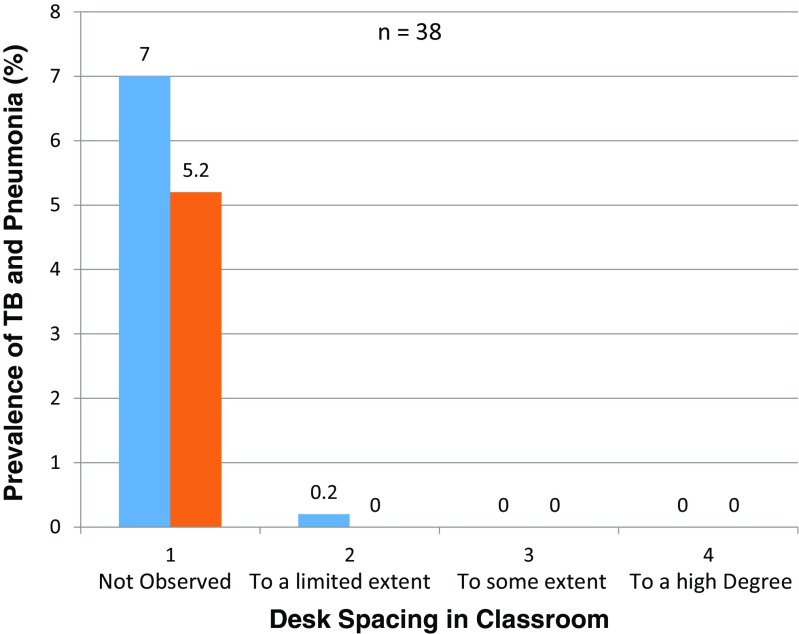
TB and pneumonia prevalence rates against desk spacing in classrooms among secondary schools in Kisumu County, Kenya

Prevalence rates of tuberculosis and pneumonia were high at 4.8 and 5.0%, respectively, in schools where standard desk spacing in classrooms was not observed and almost zero percent in schools that observed standard bed spacing. Desk spacing in classrooms and prevalence rates of tuberculosis and pneumonia had a significant association (X^2^_6, 0.05_ = 8.21, Fig. 7) but there was no strong evidence that the intervention had an effect.

## Discussion

The prevalence of malaria varies widely from area to area, as has been shown by several studies in Uganda (Nankabirwa et al. 2013; Pullan et al. 2010; Kabatereine et al. 2011). The findings of these studies, which showed that in Uganda 14–64% of school-age children have parasitemia at any one time, concurs with the determined prevalence rate of malaria that we observed (Fig. 1; X^2^_4, 0.05_ = 2.458). This also concurs with the results of a study on the prevalence of malaria parasitema by Gitonga and others (2010) in 480 Kenyan schools between September 2008 and March 2010, which found an overall prevalence rate of 4%; however, this ranged from 0 to 71%. It also agrees with the findings of a study by Dai et al. (2008); Oduro et al. (2013) and others for Senegal, The Gambia, and Mauritania in which the prevalence of malaria infection in school-age children ranged from 5 to 50%.

It is very concerning that malaria prevalence among secondary school students may interfere with their educational development. The effect of malaria infection on school absenteeism has been confirmed in several studies (Leighton and Foster 1993; Brooker et al. 2000) showing that it contributes to between 17 and 54% of school absenteeism per year.

Studies by Lengeler (2004) and Lim et al. (2011) have shown there is strong evidence that, at the individual level, regular use of an insecticide-treated net (ITN) or long-lasting insecticide-treated nets (LLIN) substantially lowers the risk of malaria infection. This may be attributed to the fact that as children become older and more independent, parents have less control over the time when they go to bed, where they sleep and whether they use a net, frequently resulting in low net coverage in children in this age group. However, evidence is needed to concretize the reasoning.

This study has revealed that student age is a predisposing factor to infectious diseases (Fig. 6). The same results were observed in a study by Baker et al. (2013) on infectious diseases attributable to household crowding in New Zealand, which showed that children less than 5 years old exposed to greater household crowding had 1.69 times the odds of pneumonia as children exposed to the least crowding. The findings of this study showed that the prevalence of tuberculosis and pneumonia is high among students where there is overcrowding in hostels and classrooms (Figs. 2 and 7). It also revealed that students in the 14–17-year age bracket have a higher incidence of tuberculosis and pneumonia than those in the 18 years and greater age bracket^16^. Findings by the CDC (2007) also confirm these results.

The World Health Organization (WHO 2007), while addressing sex and gender in epidemic-prone infectious diseases, found that differences between males and females arise because gender-based roles, behavior and power.

For most infectious diseases (Fig. 4), the difference in prevalence rates between males and females is more likely to be due to differences in exposure than to differences in immunity. For example, in many societies females spend more time at home than males during the day and therefore experience greater daytime household exposure to infections, for instance caring for the sick and changing baby diapers, than males.

Male students, in their normal lives, spend more time outside the households in the evening than female students, so they are more exposed to mosquito bites than females. This study has revealed that the prevalences of malaria, tuberculosis, pneumonia and other respiratory tract infections are lower among female than male secondary students.

For reasons that are not well understood, a study by WHO (2003) found that females had lower mortality rates from severe acute respiratory syndrome than males, a pattern that was maintained after adjusting for age. Despite the scarcity of information, there are strong indications that sex and gender are necessary for the transmission and control of epidemic-prone diseases.

Age of secondary school students is a significant vulnerability factor for malaria, diarrhea, tuberculosis and pneumonia, which were the important communicable diseases most prevalent among secondary school students in Kisumu County, Kenya.

## Footnotes

1 Nyanza is an administrative region in Kenya comprised of Kisumu and five other Counties.

2 Kisumu West is one of the sub-counties in Kisumu County.

3 Index cases were students with clinically confirmed communicable disease illnesses.

4 Level of education of respondent

5
*p* < 0.05; X^2^_5, 0.05_ = 252.672

6
*p* = 0.05 at 95%confidence interval; dF = 3.033
